# Simultaneous transmission and reception on all elements of an array: binary code excitation

**DOI:** 10.1098/rspa.2018.0831

**Published:** 2019-05-08

**Authors:** Julio A. Isla, Frederic B. Cegla

**Affiliations:** Imperial College London, London

**Keywords:** coded sequences, pulse compression, pulse echo, large arrays, low signal-to-noise ratio, ultrasound

## Abstract

Pulse-echo arrays are used in radar, sonar, seismic, medical and non-destructive evaluation. There is a trend to produce arrays with an ever-increasing number of elements. This trend presents two major challenges: (i) often the size of the elements is reduced resulting in a lower signal-to-noise ratio (SNR) and (ii) the time required to record all of the signals that correspond to every transmit–receive path increases. Coded sequences with good autocorrelation properties can increase the SNR while orthogonal sets can be used to simultaneously acquire all of the signals that correspond to every transmit–receive path. However, a central problem of conventional coded sequences is that they cannot achieve good autocorrelation and orthogonality properties simultaneously due to their length being limited by the location of the closest reflectors. In this paper, a solution to this problem is presented by using coded sequences that have receive intervals. The proposed approach can be more than one order of magnitude faster than conventional methods. In addition, binary excitation and quantization can be employed, which reduces the data throughput by roughly an order of magnitude and allows for higher sampling rates. While this concept is generally applicable to any field, a 16-element system was built to experimentally demonstrate this principle for the first time using a conventional medical ultrasound probe.

## 1. Introduction

The use of phased arrays is ubiquitous in the fields of radar, sonar, medical and industrial ultrasound [1–4]. There has always been a quest for arrays with a greater number of elements so that more information can be gathered about the medium. While the authors are from a background in the field of NDE (non-destructive evaluation), where arrays are used for the detection and characterization of defects in engineering components [5,6], the trend for increasing numbers of array elements to form better images is also illustrated by developments in the field of medical ultrasound. For example, the number of array elements has soared from a few hundred to thousands, e.g. as in the X6-1 xMATRIX array transducer (Philips Medical Systems, Andover, MA, USA) and the system reported by Gennisson *et al.* [1], the main aim being to use dense two-dimensional matrix probes to increase the quality of three-dimensional images [7–11].

This trend gains more attention as better and denser probes are developed [12–21], mainly due to the recent advances in micro-machined ultrasonic transducer (CMUT) technology. Meanwhile, progress is also being made on the instrumentation towards the full control of every array element [22] and higher probe-electronics integration [9,23–29]. For example, in [30], an array that has a density of 1061 elements mm^−2^ and operates at a central frequency of 18.6 MHz was fabricated using piezoelectric micro-machined ultrasonic transducers (PMUTs).

Despite these achievements, the full capability of dense, high-element-count probes is yet to be realized because (a) they have smaller elements, which produce lower intensity signals, and (b) the number of independent transmit-receive element pairs that can be processed is limited by the overall duration of the measurement or the desired frame rate. Increasing the number of independent transmit–receive pairs is important because it helps to increase the overall resolution. Currently, the use of a large number of independent transmit–receive pairs is mainly limited by the waiting time required between excitations to avoid interference. Since this is a limitation imposed by physics, it is equally relevant in the medical field [23], NDE [31] and other fields that use elastic or other types of waves for interrogation.

Although all of the transmit–receive pairs in an array are rarely or never used simultaneously, due to several instrumentation and processing limitations, other alternatives have been developed to increase the resolution of the resulting image. For example, row–column combination of transmit and receive elements [15,28], where the rows of the array act as transmitters and the columns as receivers. Even though this reduces the total number of independent elements to be addressed, the quality of the resulting image is suboptimal.

Plane-wave excitation is also widely used to increase the transmitted power and resolution [1,32–36], whereby plane waves are transmitted using different inclination angles; the greater the inclination angle and the smaller the angle steps are, the greater the resolution and the lower the unwanted side lobes will be. Plane-wave excitation can be used with very fast acquisition systems. This led to two recent key achievements in ultrasound technology, namely fast sub-wavelength resolution [37], though this requires the use of invasive contrast agents (see also [38]), and the study of viscoelastic properties of tissue through shear wave imaging [39].

However, in general, the number of plane waves with different angles that can be transmitted is limited by the waiting time required between transmissions and therefore this technique is less time-efficient for three-dimensional imaging, where the number of plane waves with different inclination angles increases due to the third dimension [1,40]. Moreover, plane waves generated by finite apertures are just approximations constrained to a limited region under the aperture and to a maximum number of inclination angles; outside this region, the single wavefront approximation, necessary for adequate resolution, breaks down.

The use of sparse arrays, as an alternative to using a large number of independent transmit–receive pairs, is another technique that provides an increase in resolution with moderate grating lobes, whereby fewer elements are sparsely distributed with a mean distance between the elements greater than that required to avoid grating lobes [41–45]. In general, sparse arrays have overall greater grating lobes than arrays that have equivalent area but a greater number of elements [46], which results in higher interference from reflectors outside the focal zone.

Another key factor to consider when dealing with dense arrays is the signal-to-noise ratio (SNR). Dense array probes are expected to have smaller elements, which radiate and receive less power, and therefore the SNR has to be increased by, for example, using pulse-compression techniques, such as coded sequences that have good autocorrelation properties; good autocorrelation properties are required to minimize the noise introduced by the sequences. On the other hand, orthogonal sequences can speed up the number of transmit–receive pairs that can be processed in parallel. In the last decades, a tremendous effort has been placed in the development of sequences which have both good autocorrelation and orthogonality properties [47–52]. Most of this effort has been stemmed by the field of communication technology with development of techniques such as in code division multiple access (CDMA).

One central problem in the use of conventional coded sequences is that a single set of coded sequences cannot achieve good autocorrelation and orthogonality properties simultaneously. However, it is essential to highlight the work reported in [47], wherein complementary sets of sequences that achieve perfect autocorrelation and orthogonality were introduced. The work reported in [47] extended the complementary principle introduced in [53] to orthogonal channels. It is also worth mentioning that an alternative to complementary sequences is the use of zero-autocorrelation-zone sequences [49,54–57]. These sequences only achieve perfect autocorrelation within a given interval, which may suffice in many practical applications, and therefore some constraints in the construction of the sequences can be relaxed. Zero-autocorrelation-zone sequences are mainly reported for single channel applications, but from the authors' point of view, there is no reason, other than the computational burden, that prevents this methodology from being used for orthogonal multi-channel systems. Further discussions on the use of coded sequences in ultrasound can be found in [58–62].

**Figure 1.**
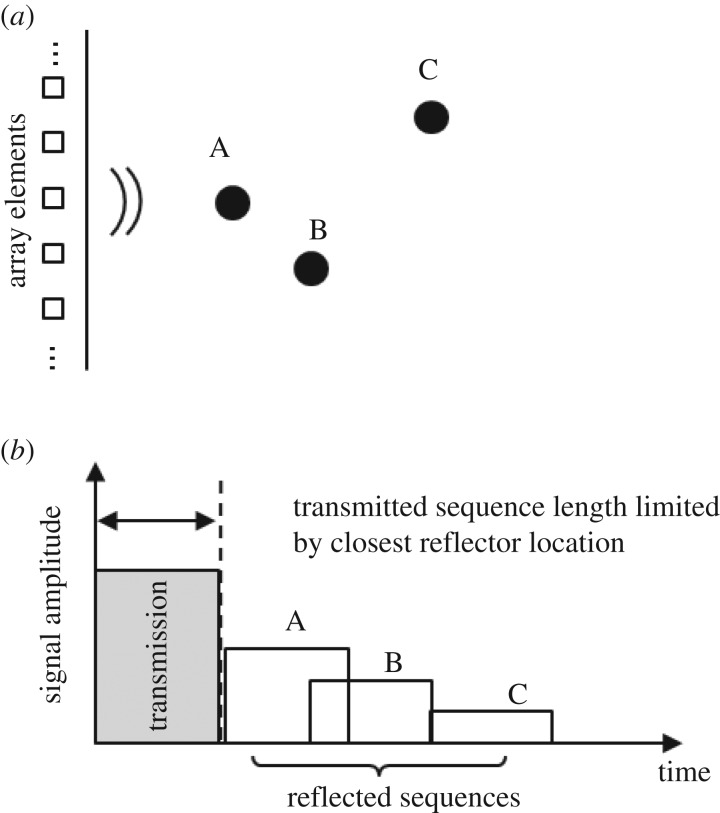
A transducer operating in pulse-echo mode (*a*) and the corresponding received signals (*b*). The length of the excitation is limited by the location of the closest reflector A (A, B and C illustrate point-like reflectors).

In spite of all of the progress made related to the synthesis of sequences which have good autocorrelation and orthogonality properties, there is still a central drawback: the sequences have to be long in order to achieve sufficient orthogonality between the channels and SNR increase, but in ultrasound imaging, the length of the sequences is limited by the distance between the probe and the closest reflectors [61,62]. This problem is illustrated in figure 1*a*,*b*.

In this paper, the authors extend their previous work on single-channel, pulse-echo coded sequences [63–65] to multi-channel systems by introducing a set of sequences which have intervals where reception can take place. As a result, the information of every transmit–receive pair can be acquired simultaneously while the overall length of the sequences is no longer limited by the location of the closest reflectors. This is an important result because there exist many applications where the SNR on reception is intrinsically low [66–74] and a substantial SNR increase is required by means of coded excitation. Moreover, the proposed sequences could enable a new disruptive design paradigm whereby dense array probes are designed such that their elements radiate less energy, thereby relaxing many design constraints while enabling higher low-power electronics integration.

An advantageous consequence of using low-SNR arrays is that a significant simplification of the electronics and a reduction in data throughput are possible. This is because when the received signals lie close to or below the noise threshold, analogue-to-digital converters (ADCs) can be replaced by comparators with a negligible (2-dB) decrease in SNR [75–78]. In [78], the authors investigated the maximum possible dynamic range of binary quantization under different conditions and found that binary quantization can be used when the input SNR is lower than 5 dB. Comparators can also operate at higher sampling rates, which is particularly useful for high-frequency arrays [14,24]. Furthermore, binary excitation combined with pulse-width modulation has been used to control the shape of the transmitted waves with similar performance to that of digital-to-analogue converters (DACs) [79].

**Figure 2.**
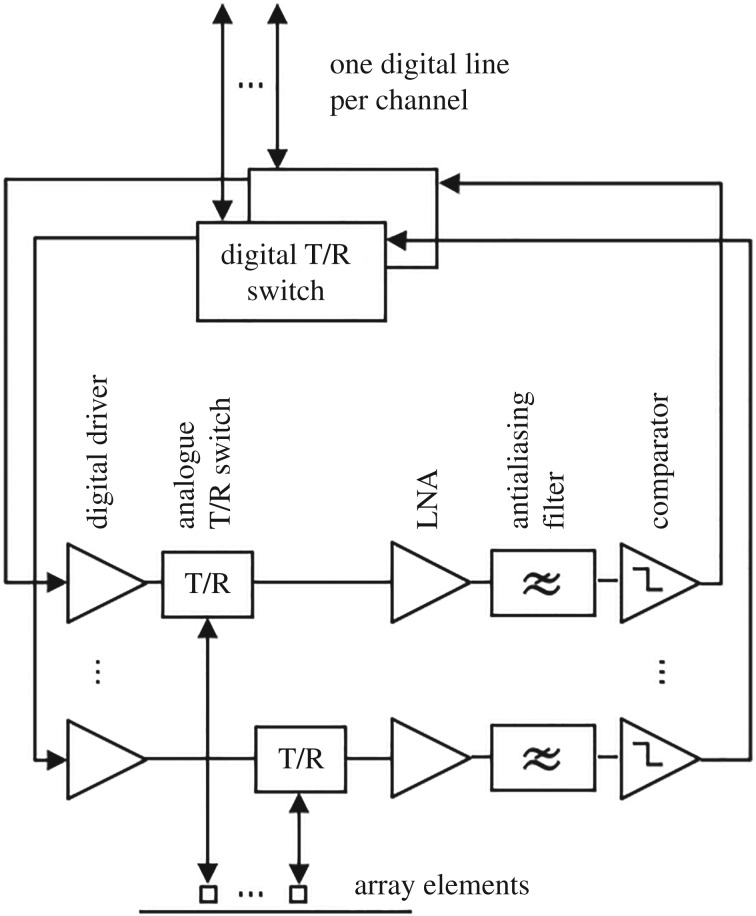
Proposed system architecture to reduce data throughput. Each array element is fully controlled by a digital line. ADCs can be replaced by comparators because the received signal is close to or below the noise threshold. LNA stands for low-noise amplifier.

Leveraging the use of binary excitation and quantization, this paper introduces the first fully binary pulse-echo array controller wherein each element is solely addressed by one digital line (figure 2). This new architecture is to be used with the pulse-echo coded sequences that are also introduced in this paper so that simultaneous transmission and acquisition of data is enabled. The performance of the sequences is derived theoretically and validated through simulations of simple set-ups. We have also built a 16-channel array controller; experimental results from simple wire and spring targets are presented to corroborate the theoretical results using this controller.

The organization of this paper is as follows. First, there is a background section to recall synthetic image focusing and single-channel coded excitation with receive intervals. Then, a multi-channel sequence set with receive intervals is introduced along with the formulae for the resulting SNR of the focused image. Following that, simulations are carried out to investigate the performance of the proposed sequences under different scenarios. Later, we report the performance of the 16-channel binary array controller. Finally, the conclusions are presented after a discussion of the results.

## 2. Background

In this section, single-channel coded excitation with receive intervals is recalled. This was previously introduced by the authors in [63–65]. Synthetic array focusing using every transmit–receive path is also revisited. These techniques are cornerstones of the methodology presented in this paper.

### (a) Single-channel random coded excitation with receive intervals

In a pulse-echo set-up, the maximum length of standard transmitted sequences is limited by the location of the closest reflector. In figure 1*a*,*b*, it can be seen that if the transmit length is extended any further, then information from the closest reflector will be lost as the transmitter would still be operating and so could not switch to receive mode. In order to circumvent this problem, receive intervals are introduced in the sequence so that reception can take place [63–65].

A sequence with receive intervals can be obtained as the multiplication of two sequences, one that controls the transmit and receive intervals, which takes on values 0 (receive) and 1 (transmit), and another that controls the polarity of the transmitted bursts, which takes on values 1 and −1. The resulting sequence then takes on values 1, −1 or 0, where 0 indicates that reception takes place.

The SNR obtained when cross-correlating the received and transmitted sequences is (see [63–65])

2.1 $${\text{SNR}}_{\mathrm{gaps}}\approx \frac{\left(1-p_{1}\right)L}{1+(1/p_{1}{\text{SNR}}_{\mathrm{in}})},$$

where SNR_in_ is the input or received SNR, *L* is the total number of receive and transmit intervals and *p*_1_ is the ratio of the number of transmit intervals to the total number of intervals in the sequence. Equation (2.1) corresponds to the case where a transmit interval consists of a delta function.

### (b) Synthetic aperture focusing using every transmit–receive path

**Figure 3.**
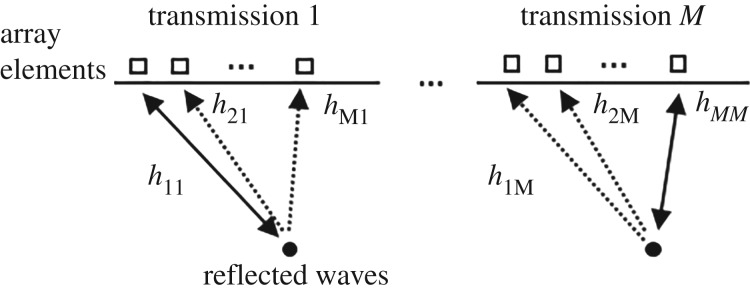
Standard acquisition of every transmit–receive pair. Only one element act as a transmitter at a time with all of the elements subsequently switching to receive mode in parallel.

The ability to focus the ultrasonic waves on transmit and receive increases the resolution and contrast at a cost of extra processing. The total focusing method (TFM) [3] is the form of synthetic aperture focusing usually known in the non-destructive evaluation (NDE) community. To implement it, signals from every transmit–receive path are acquired. This process is shown in figure 3, where, for example, *h*_21_ is the path from transmit element 1 to receive element 2.

In the two-dimensional case, the goal is to form a focused image *I*_*xy*_, where *x* and *y* are the coordinates of the image. To accomplish this, each received signal is delayed following a focal law so that they add up coherently at the coordinate of interest

2.2 $$I_{xy}=\sum_{a}\sum_{b}h_{ab}^{\prime }\left(t\right)\cdot d_{abxy}\left(t\right)$$

and

2.3 $$d_{abxy}\left(t\right)=\gamma_{ab}\delta \left[t-\frac{\sqrt{{\left(x_{a}-x\right)}^{2}+y^{2}}+\sqrt{{\left(x_{b}-x\right)}^{2}+y^{2}}}{c}\right],$$

where *h*′_*ab*_ is the signal that corresponds to the *h*_*ab*_ path, *a* and *b* are the indices of the receive and transmit elements, *γ*_*ab*_ is a constant, which can be used, for example, to create apodization effects, *c* is the speed of the wave, *δ* is a delta function and *t* corresponds to time. Equation (2.2) can readily be extended to the three-dimensional case where a volume is obtained instead of an image. Also, the Fourier transform can be employed to significantly speed up the computation [3,80–82]. Further processing techniques have also been investigated with the aim of increasing the quality of the focused image [83,84].

## 3. Principle of operation and performance of synthetic aperture focusing using a random sequence set that has receive intervals

In this section, a set of random sequences that have receive intervals and can be used to simultaneously excite each of the array elements is introduced. The received signals are cross-correlated with the transmitted sequences to recover the signals that correspond to every transmit–receive path.

### (a) System overview

**Figure 4.**
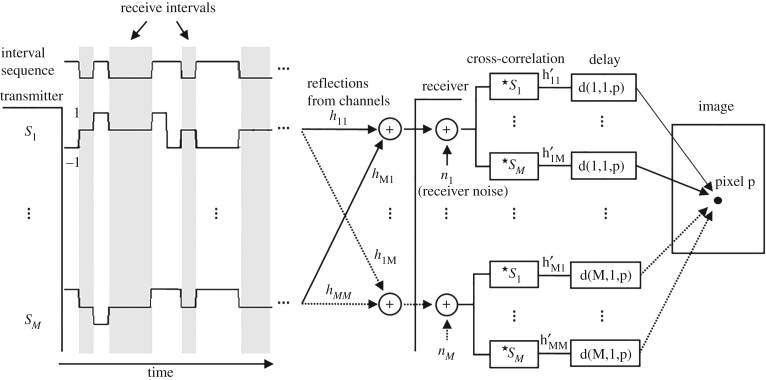
Synthetic focusing using a pulse-echo system with a random set of sequences which have receive intervals. Note that the sequence that controls whether transmission or reception takes place is common to all of the channels.

Figure 4 shows an overview of the proposed system. On the left-hand side of the figure, *M* transmitters are excited at the same time. A different sequence (*s*_1_, *s*_2_, …, *s*_*M*_) is used in each channel. The sequences have the same amplitude and number of receive intervals and are uncorrelated. These sequences take on values 1, −1 or 0, and can be obtained by multiplying two random binary sequences: one common sequence to every channel, which controls the occurrence of the transmit and receive intervals and takes on values 1 (transmit) and 0 (receive); and another that is different for each channel and takes on values 1 and −1 to control the polarity of the bursts. The grey stripes in figure 4 correspond to the receive intervals. To simplify the analysis, the bursts are considered to be delta functions; this is extended to any type of burst in §§3d.

By using this system, the signals (*h*′_11_, *h*′_12_, …, *h*′_*MM*_) that correspond to each of the *M*^2^ paths (*h*_11_, *h*_12_, …, *h*_*MM*_) can be recovered from the *M* transmitted sequences, albeit with an added amount of noise, interference or error. In practice, there is a noise source associated with each receiver input (*n*_1_, *n*_2_, …, *n*_*M*_) due to the receive electronics and the transducer. These noise sources are uncorrelated to one another and to the sequences. The signals *h*′_11_, *h*′_12_, …, *h*′_*MM*_ are recovered by cross-correlating the received signals with the initially transmitted sequences; the operator {⋆} in the figure stands for cross-correlation. Finally, once these signals are recovered, they are delayed according to equation (2.2) to produce a focused image. The overall process is summarized in the box Algorithm 1.


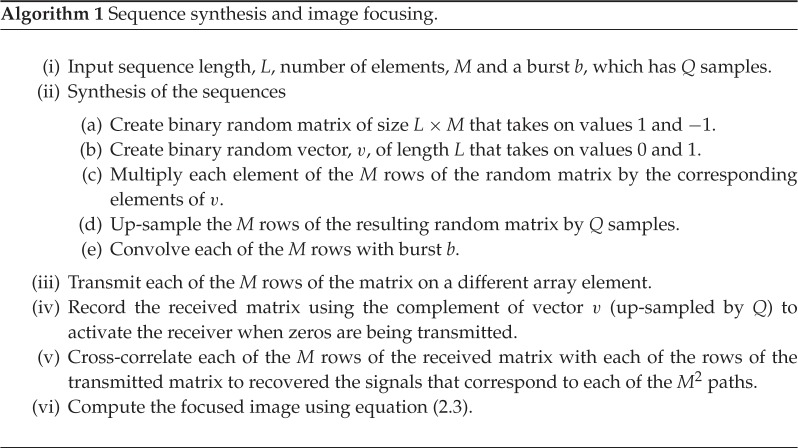


### (b) Signal-to-noise ratio of the focused image

Based on equation (2.1), the expected SNR of each recovered signal that corresponds to a given path is

3.1 $${\text{SNR}}_{h}\approx \frac{(1-p_{1})\;L}{M+(1/p_{1}{\text{SNR}}_{\mathrm{in}})},$$

where SNR_in_ is the ratio of the power of one isolated received sequence to a receiver noise source, *M* is the number of array elements, *L* is the total number of receive and transmit intervals and *p*_1_ is the ratio of the number of transmit intervals to the total number of intervals as in equation (2.1). For simplicity, all of the received sequences are assumed to have the same amplitude. The sequence noise, given by the left-hand term of the denominator, increases from 1 to *M* with respect to equation (2.1) due to the interference of the extra *M* − 1 sequences that are combined at the receiver input.

Once the signals that correspond to all of the *M*^2^ paths are recovered, they are delayed according to equation (2.2) to obtain the focused image. It is assumed that no path is the same. Therefore, the received sequences that travelled through different paths are uncorrelated as well as any noise source associated with them, except at the focal point. For this step, note that cross-correlation is not commutative when the inputs have no symmetry, i.e. *s*_*a*_⋆*s*_*a*′_≠*s*_*a*′_⋆*s*_*a*_. Finally, the SNR of the image of a single point-like reflector can be defined as the ratio of the energy at the focal point to the variance of a region in the image that does not include the focal point or any focusing artefacts. Since signals from *M*^2^ paths are combined, the resulting SNR is

3.2 $${\text{SNR}}_{I}\leq \frac{\left(1-p_{1}\right)L\cdot M}{1+(1/p_{1}{\text{SNR}}_{\mathrm{in}}M)}.$$

The inequality symbol in equation (3.2) indicates that this is an upper bound for SNR_I_. In practice, this upper bound is difficult to reach due to the coherent interference caused by the focusing algorithm, namely the point spread function (PSF), and the unequal intensity of the signals corresponding to each of the paths. The latter can be due to one or more of the following issues: (a) some adjacent elements may have the same path, mainly because time and spatial dimensions are discretized, therefore violating the independence assumption; (b) elements further from the reflector receive less intense signals; (c) elements and reflectors are non-isotropic and therefore the intensity of the received/transmitted waves varies with the orientation of the reflectors relative to the elements; (d) paths from different reflectors may interfere with one another; and (e) direct wave incidence from transmitting elements that propagates through the array probe or along the surface of the specimen can be seen as coherent interference.

When SNR_in_≪(2/*M*) and *p*_1_ = 0.5, equation (3.2) simplifies to

3.3 $${\text{SNR}}_{I}{|}_{{\mathrm{SNR}}_{\mathrm{in}}\ll \frac{2}{M},p_{1}=0.5}\leq 0.25L\cdot M^{2}{\text{SNR}}_{\mathrm{in}}.$$

In this regime, the noise introduced by the receive electronics is greater than that of the sequences themselves. Hence, the use of any sequences more sophisticated than a random sequence with *p*_1_ = 0.5 will not have a substantial effect on the results and the random sequences can be said to be optimal in this case.

This leads us to a last observation where we should note that we impose no constraint on *L* or *M*, and this could also include the extreme case where *L* = 1, which corresponds to the fastest performance possible. This means that we accept that interference between the paths occurs, i.e. channels are not strictly orthogonal, but treat this as a noise source, just like the noise from the receive amplifiers, and focus on maximizing the image SNR by increasing *L* and *M*; the optimal choice of *L* and *M* is addressed in the next section. Note that this is different from the more conventional approach where perfect orthogonality is sought, i.e. where the recovered signals corresponding to each one of the possible paths are free from interference from one another and hence the number of coded symbols per channel has to be equal to or greater than the number of channels themselves; the closest analogy to the proposed sequences would be *p*_1_*L*≥*M*.

### (c) Comparison with systems that do not use coded excitation

In this section, we discuss the expected SNR when using *N* sequential transmit intervals per channel (only one transmit interval per channel is fired at a time) and average them so that the overall duration including receive intervals is equal to *L*; see [65] for the single-channel case. In the presence of *M* channels, the resulting SNR is

3.4 $${\text{SNR}}_{\mathrm{ave}}=N\cdot M^{2}{\text{SNR}}_{\mathrm{in}}.$$

Let the ratio between the total number of sequential transmit intervals and the total number of transmit and receive intervals be

3.5 $$r=\frac{N\cdot M}{L}.$$

Note that when firing sequential transmit intervals, at least *M* transmit intervals and *M* receive intervals are required. So, to establish a fair comparison with a coded sequence of equivalent duration, *L*≥2*M* and hence *r* ≤ 0.5. Finally, the ratio between the SNR using coded excitation and sequential firing is

3.6 $$\alpha =\frac{{\text{SNR}}_{I,\max}}{{\text{SNR}}_{\mathrm{ave}}}=\frac{\left(1-p_{1,\max}\right)}{r\left({\text{SNR}}_{\mathrm{in}}+(1/p_{1,\max}M)\right)},$$

where *p*_1,max_ is the value of *p*_1_ that maximizes SNR_I_. Of particular, interest is the case

3.7 $${\alpha |}_{{\mathrm{SNR}}_{\mathrm{in}}\ll \frac{2}{M},p_{1,\max}=0.5}\approx \frac{M}{4r}\geq 1,$$

where, since *r* ≤ 0.5 and *M*≥2, the proposed sequence set outperforms sequential firing irrespective of *r* and *M*.

**Figure 5.**
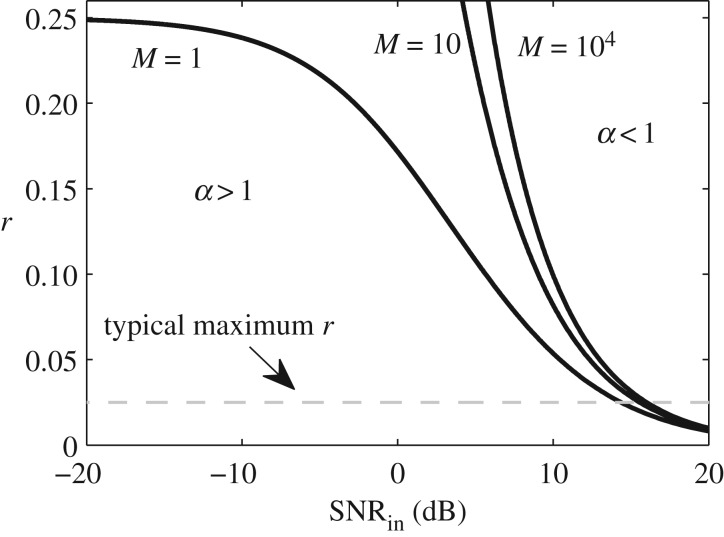
Ratio between sequential transmit intervals and total number of intervals, *r* = (*N* · *M*/*L*), versus input SNR, SNR_in_, for which the SNR obtained when using the proposed sequences set and sequential firing and averaging is the same, i.e. *α* = 1. Any combination of *r* and SNR_in_ below the curves implies that *α* > 1, and hence the proposed sequence set yields a greater SNR. The dashed grey line corresponds to *r* = (1/40), which is at the high end of what is expected from typical pulse-echo ultrasound systems.

Figure 5 shows *r* versus SNR_in_ subject to *α* = 1 for *M*∈{1, 10, 10^4^} (continuous curves). Any combination of *r* and SNR_ in_ that lies below the curves implies that *α* > 1, and hence the proposed sequence set yields a higher SNR. Note that once *M* > 10 the behaviour of the curves is not substantially different. As a rule of thumb, once SNR_ in_ < 10 dB, the proposed sequence set can be said to outperform sequential firing. It was found that *p*_1,max_ ≈ 0.5 in all of these cases.

The dashed line in figure 5 corresponds to a typical maximum value of *r* = 0.025, equivalent to 39 receive intervals per sequential transmit interval. To give an example, say *M* = 10^3^, SNR_ in_ = − 10 dB and *r* = 0.025, then *α* ≈ 196 and therefore the proposed sequence set yields an extra 23-dB-SNR increase for the same excitation duration in comparison with sequential firing.

### (d) Signal-to-noise ratio adjusted for modulation and uneven paths

Equation (3.2) gives a quick estimate of the performance of the sequences where the transmit intervals consist of a delta function. In practice, modulation is required due to the limited bandwidth. This can be simple sinusoidal bursts or more complex chirp bursts [59,85–87]. The effect of modulation can be readily quantified by up-sampling the sequences by the relevant number of samples and convolving the result with the desired burst. After some algebra (see appendix A), it can be shown that

3.8 $${\text{SNR}}_{I,\mathrm{mod}}\leq \frac{L\left(1-p_{1}\right){\left(\sum_{i=1}^{M}\sum_{j=1}^{M}w_{ij}\right)}^{2}}{\sigma_{b}^{2}M\sum_{i=1}^{M}\sum_{j=1}^{M}w_{ij}^{2}+(M^{2}/p_{1}{\text{SNR}}_{\mathrm{in}})},$$

where *w*_*ij*_ is the amplitude of the received echo from transmitter *i* and receiver *j*, and *σ*^2^_*b*_ is the variance of the modulated burst, which is assumed to have mean zero. SNR_in_ is the input SNR with respect to the strongest reflection. Equation (3.8) is just an upper bound for SNR_I,mod_ due to the effect of coherent noise. Note that if *w*_*ij*_ = 1, ∀ *i*, *j*, and *σ*^2^_symb_ = 1, equation (3.8) reduces to (3.2).

Equation (3.8) corresponds to the case where the received signal is cross-correlated with the unmodulated sequences. The effect of matched filtering, i.e. cross-correlating the received signal with modulated sequences whose symbols are not delta function but the actual transmitted symbols, was empirically found (see [65]) to be

3.9 $${\text{SNR}}_{I,\mathrm{mod}}^{\prime }\leq \frac{L\left(1-p_{1}\right){\left(\sum_{i=1}^{M}\sum_{j=1}^{M}w_{ij}\right)}^{2}Q\sigma_{b}^{2}}{2Q\sigma_{b}^{2}\sigma_{bb}^{2}M\sum_{i=1}^{M}\sum_{j=1}^{M}w_{ij}^{2}+(M^{2}/p_{1}{\text{SNR}}_{\mathrm{in}})},$$

where *Q* is the number of samples of the burst and *σ*^2^_*bb*_ is the variance of the normalized autocorrelation of the burst.

In the case of a point-like reflector, the coefficients *w*_*ij*_ can be obtained by simple algebra taking into account the effect of beam spread and element directivity [3]. For example, in the two-dimensional case, the losses due to beam spread with respect to the *i*^th^ array element are

3.10 $$\beta_{i}=\sqrt{\frac{y^{\prime }}{\sqrt{{\left(x_{i}-x^{\prime }\right)}^{2}+{\left(y^{\prime }\right)}^{2}}}},$$

where *x*′ and *y*′ are the locations of the reflector with respect to the linear array. The role of *y*′ in the numerator is to normalize *β*_*i*_ to the distance between the reflector and the linear array. In this particular case, it can be shown based on [88] that *β*^2^_*i*_ is an acceptable approximation to the directivity of point-like array elements, assuming that the main acceleration component of the array elements is normal to the array/specimen surface. Then, *w*_*ij*_ = (*β*_*i*_*β*_*j*_)^3^.

## 4. Numerical simulations

**Figure 6.**
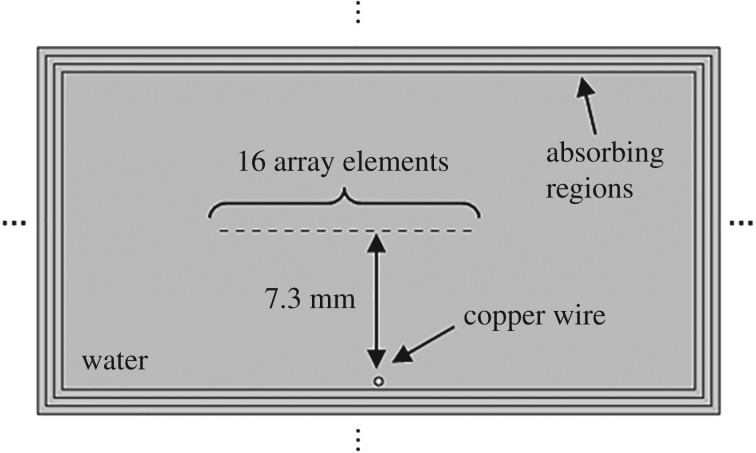
Finite-element model.

In this section, the performance of the sequences is investigated by simulating a 16-element array using the Pressure Acoustic and Solid Mechanics (transient) modules of COMSOL Multyphysics 5.2 (COMSOL Inc., MA, USA). Figure 6 shows the two-dimensional finite-element model used. Each element consists of a line, which has a length equal to a quarter of the wavelength at 1 MHz, spaced by half a wavelength (from the centre of the element). The medium is simulated as water and a circle which has a diameter equal to a quarter of the wavelength is placed 7.3 mm beneath the middle of the 10th and 11th elements. The circle is used to simulate a significant acoustic impedance change in the medium, e.g. water–copper.

Absorbing layers were placed at the boundaries to attenuate any reflections by more than 40 dB. The use of the absorbing boundaries is essential because otherwise an impractically large model would have been required to prevent the reverberation of the long sequences that will be simulated. The absorbing layers were simulated in a similar way as described in [89]. Sixteen absorbing layers were employed, the most inner one had a width of a quarter of a wavelength and this was increased following a quadratic law so that the outer layer was half-a-wavelength wide. In each absorbing layer, the dynamic and bulk viscosity were increased following a cubic law so that the value of the outer region was 200 Pa s. Free-triangular elements were used for the mesh. By setting the maximum size of the elements to one-sixth of the wavelength, the errors in the focused image are not greater than 1% with respect to its maximum value. This value was selected by the authors as an acceptable compromise between accuracy and simulation time.

In an initial simulation, each array element was sequentially excited using a three-cycle burst which has a Hann apodization and a central frequency of 1 MHz. The Hann apodization is important to reduce the sidelobes of the excitation spectrum and therefore aliasing. A sampling frequency of 16 MHz was employed. The excitation was simulated as the normal component of the acceleration along the line that defines the element. For each excited element, the received signals corresponding to all of the elements were recorded, which resulted in a total of 16^2^ signals. The received signals were obtained as the added normal acceleration (with respect to the line that defines the element) of the end points of the lines that defined the elements.

**Figure 7.**
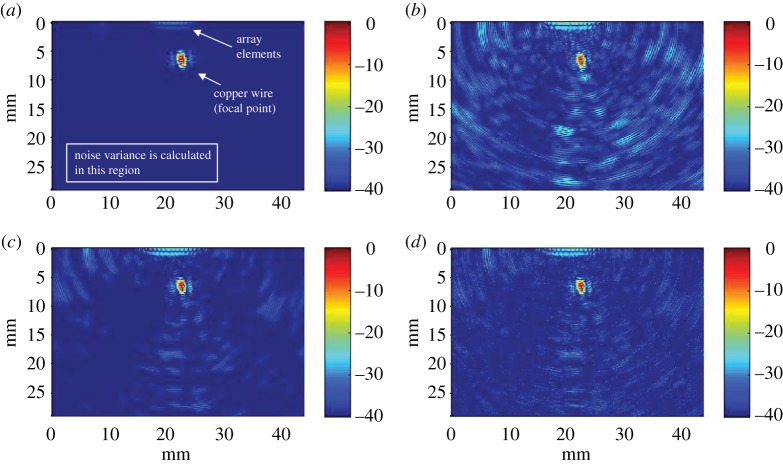
Focused images. (*a*) Elements excited one at a time; no sequences used, SNR_ in_ = ∞ dB. (*b*) Sequence that has *L* = 250 and SNR_in_ = ∞ dB. (*c*) Sequence that has *L* = 10^3^ and SNR_in_ = ∞ dB. (*d*) Sequence that has *L* = 10^3^ and SNR_in_ = 0 dB. Colour scale is given in dB. (Online version in colour.)

Equation (2.2) was employed to produce a focused image using a regular spatial grid with a resolution equal to one-sixteenth of the wavelength. The absolute value of the result is shown in figure 7*a*. The circle that corresponds to the area simulated as copper can be easily identified. Note there are no sidelobes or focusing artifacts greater than −40 dB.

The simulations were repeated using the proposed sequence set. Sets of *M* = 16 random sequences with length *L*∈{250, 10^3^} and *p*_1_ = 0.5 were generated. Each sequence in the set was up-sampled so that the transmitted burst has a length of 4 μ*s*. Then the sequences were convolved with a three-cycle burst that has a Hann apodization and a central frequency of 1 MHz. This left a 1 μs guard interval to allow any ringing from the excitation to die out before the receive intervals start; this is due to the fact that simulations are band-limited and therefore ringing may occur.

The normal acceleration was recorded at each element location. The 16 received signals were cross-correlated with the original non-modulated (but up-sampled) sequences. The modulation was omitted to avoid the effect of matched filtering so that the SNR from theoretical and experimental results can be more easily compared. By doing so, the signals corresponding to the 16^2^ possible paths were recovered. Figure 7*b*,*c* shows the absolute values after applying the focusing algorithm.

Note that from figure 7*a*, we can conclude that the predominant noise in figure 7*b*,*c* is due to the sequence noise, and that this is an order of magnitude greater than the coherent noise of the PSF of the copper reflector or the absorbing boundaries. Having the noise of the sequence as the predominant source of noise is a necessary condition to use equation (3.8). At the end of this section, we will compare the simulations with the theoretical prediction of equation (3.8).

To simulate the effect of the receiver noise on each of the array elements, a vector of normally distributed values with mean zero was added to each of the 16 received signals in the set where *L* = 10^3^; a different vector was used for each signal. Note that wide-band noise is used; its frequency content is not relevant in this case because no filtering will be done. The variance of the noise vectors was selected so that the input SNR was SNR_in_ = 0 dB with respect to the peak value of the reflection from the area simulated as copper to the closest transducer. Results from the simulation are shown in figure 7*d*. It can be appreciated that adding noise which has a variance equivalent to the signal peak value, i.e. SNR_ in_ = 0 dB, does not significantly affect the quality of the image compared to figure 7*c*. This is because the noise of the sequences predominates.

To compare the results of the simulations with those predicted by equation (3.8), the SNR of the focused image was calculated for the cases where SNR_ in_∈{∞, 0, − 10, − 20} dB, *L*∈{250, 500, 10^3^} and *p*_1_ = 0.5. In each case, the image SNR, SNR_I,mod_, was calculated as the inverse of the variance of the image in the region shown in figure 7*a*, where there are no reflections or focusing artefacts, having previously normalized the image by the peak amplitude. In calculating equation (3.8), the variance of the burst was computed for the strongest received echo giving *σ*^2^_*b*_ = 0.2. This corresponds to a 7-dB increase with respect to the ideal case of equation (3.2) for the cases where the sequence noise dominates the electronics noise. The sums of the weights *w*_*ij*_ were 0.55 for the numerator and 0.57 for the right-hand term of the denominator of equation (3.8).

**Figure 8.**
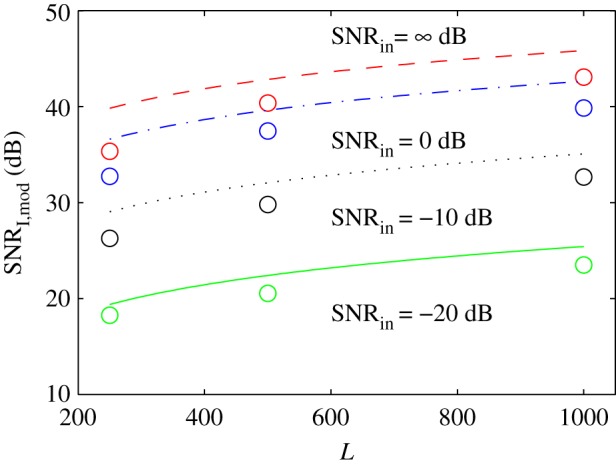
Predicted upper bound of equation (3.8) (curves) and simulated image SNR (circle markers) using *M* = 16 elements. (Online version in colour.)

The predicted and simulated results are compared in figure 8. The curves correspond to the predicted upper bound of SNR_I,mod_, as given by equation (3.8), for SNR_ in_∈{∞, 0, − 10, − 20} dB and *L*∈[250, 10^3^]. The circle markers correspond to the simulated results. In general, there is a slight offset (less than 3 dB) between simulated and theoretical results. This offset is expected since equation (3.8) is just an upper bound for SNR_I,mod_. The case where SNR_in_ = ∞ dB and *L* = 250 has more than a 3-dB difference with respect to the theoretical value; this is a result of the sequence not behaving completely randomly due to its relatively short length.

It should be pointed out that coding and focusing are linear and independent processes and therefore the sidelobes of the PSF are independent of the noise introduced by the sequences and the receiver. In the far field, the amplitude of these sidelobes is mainly controlled by the tapering of the aperture. This means that the sidelobes of the PSF limit how much the SNR of an image can be improved by increasing the length of the sequences. For example, in figure 7*c*,*d*, a further increase in the length of the sequence, *L*, will rapidly lead to the situation where the sidelobes of the PSF become dominant over the noise/interference introduced by the sequences, from which point onwards, there is no value in increasing *L* any further.

In the next section, we will present experimental results using a fully binary, 16-channel array controller and a set-up similar to the one in the simulations but with a central frequency of 5 MHz and binary excitation; a central frequency of 5 MHz was chosen because a significant number of medical probes operate at this frequency. Note that simulations had to be conducted at a maximum central frequency of 1 MHz and using a non-binary excitation to limit the bandwidth due to the computing resources available to the authors. Simulations at 5 MHz would have required a denser mesh and more time steps. However, the frequency content of the signals is not a key factor for the performance of the sequences and both simulation and experiments will be shown to agree, though independently, with the theoretical predictions, which is a central objective of this paper.

## 5. Experimental results

**Figure 9.**
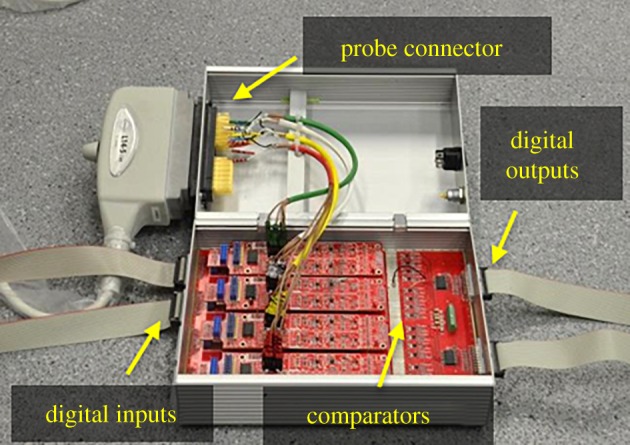
Fully binary, 16-channel array controller. (Online version in colour.)

A fully binary, 16-channel array controller was built as described in figure 2; see figure 9. Two (one receive and one transmit) binary input–output cards (M2i.7020; Spectrum Systementwicklung Microelectronic GmbH, Germany) were connected to the PCI-express bus of a computer. These cards feature 32 channels, a maximum sampling rate of 125 MHz and a maximum data throughput of 1.96 Gbit s^−1^. The transmit card controls the binary ultrasound pulsers and transmit/receive (T/R) switches (STHV748; STMicroelectronics, Switzerland). The output of the T/R switches is connected to an ultra-low-noise amplifier, which has a gain of 60 dB and a bandwidth of 10 MHz. The output of the amplifiers is connected to a comparator, and the binary output of the comparators is connected to the binary input of the receive card.

**Figure 10.**
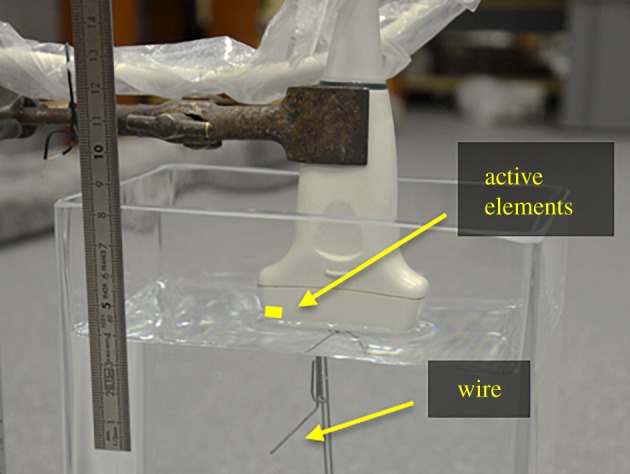
Experimental set-up. The end of the probe is submerged in water and a wire is placed underneath the probe at a depth of approximately 27 mm. (Online version in colour.)

The first 16 elements of a medical array probe (L14-5/38; Ultrasonicx, Canada), which has a bandwidth of 5–14 MHz, a focal range of 20–90 mm and an estimated pitch of 0.33 mm, was connected to the array controller. The end of the probe was submerged in water and a straight wire was placed underneath the probe at a depth of approximately 27 mm as shown in figure 10. This is to approximate a point reflector in the focal plane of the probe. The pulsers were set to transmit squared pulses of ± 4 V and a 10-Ohm resistor was connected to the input of the receivers to attenuate the signal so that the echoes at the input of the comparators were just below the noise level, i.e. SNR_ in_ < 5 dB. This is a necessary condition for binary quantization in order to produce a linear response [78].

A set of sequences was synthesized as described in Algorithm ?? using *M* = 16 random sequences with *L* = 10^3^ intervals and *p*_1_ = 0.5. The sequences were up-sampled using a sampling frequency of 40 MHz so that the transmit intervals had a length of 10 μs. As a result, the total length of the sequences was 10 ms. This is equivalent to a repetition rate of 100 Hz. Then, the sequences were convolved with a one-cycle rectangular burst centred at 5 MHz. Note that the transmit interval had a guard interval of roughly 10 μs to allow any ringing from the excitation to die out before the receive intervals start. The length of this guard interval can be reduced if active damping is employed, but this was not considered at this stage in order to simplify the system.

**Figure 11.**
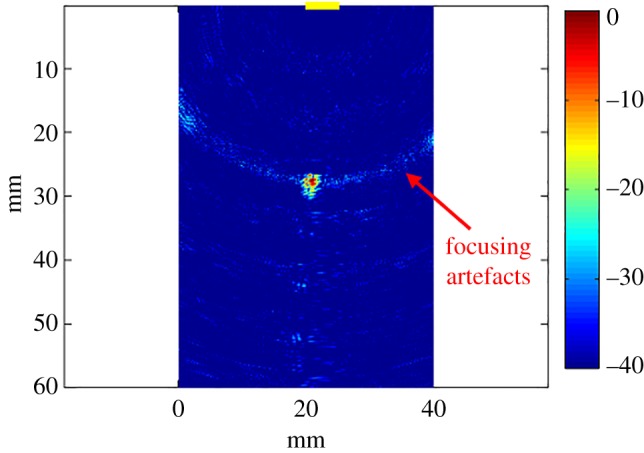
Focused image of a wire underneath the probe experimentally acquired using *M* = 16 elements and a set of random sequences that have *L* = 10^3^ intervals and *p*_1_ = 0.5. The burst consists of one rectangular cycle centred at 5 MHz. The colour scale is in dB. The array location is indicated by the yellow line on the top of the figure. (Online version in colour.)

The received signals were cross-correlated with the transmitted sequences to recover the signals corresponding to the 256 paths. The recovered signals were delayed and superimposed to form the focused image according to equation (2.2) using a regular spatial grid with a resolution equal to one-eighth of the wavelength. The resulting image, using a sequence length of *L* = 10^3^ and zeroing the first 400 samples of each signal, is shown in figure 11. The wire can be successfully identified. Focusing artefacts are highlighted in the figure; these artifacts are due to the image reconstruction algorithm and not to the sequences.

**Figure 12.**
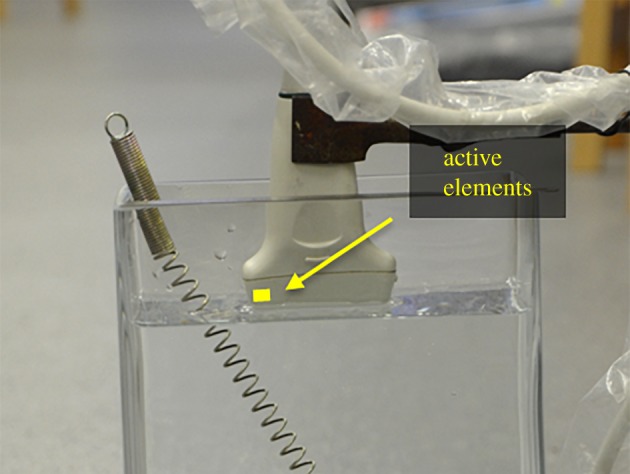
Experimental set-up. The end of the probe is submerged in water and a helical spring is placed underneath the probe along the focal plane. (Online version in colour.)

**Figure 13.**
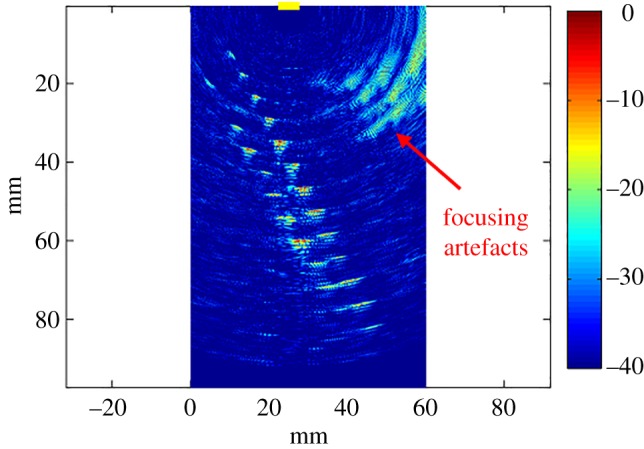
Focused image of a helical spring underneath the probe experimentally acquired using *M* = 16 elements and a set of random sequences that have *L* = 10^3^ intervals and *p*_1_ = 0.5. The burst consists of one rectangular cycle centred at 5 MHz. The colour scale is in dB. The array location is indicated by the yellow line on the top of the figure. (Online version in colour.)

To study the effect of multiple reflectors at different depths, a helical spring was placed underneath the probe as shown in figure 12. The resulting focused image is shown in figure 13; the same parameters of the previous example were used in this case. The sections of the spring that intersect the focal plane of the probe are successfully imaged. The SNR_I,mod_ is not substantially affected by the existence of multiple reflectors since it is determined by just a few dominant reflectors. The penetration depth is nearly 90 mm; this is not limited by the sequences themselves. Focusing artefacts can be observed in the top-left region of the figure; this is an undesired effect of the focusing algorithm, which is independent of the use of the sequences.

**Figure 14.**
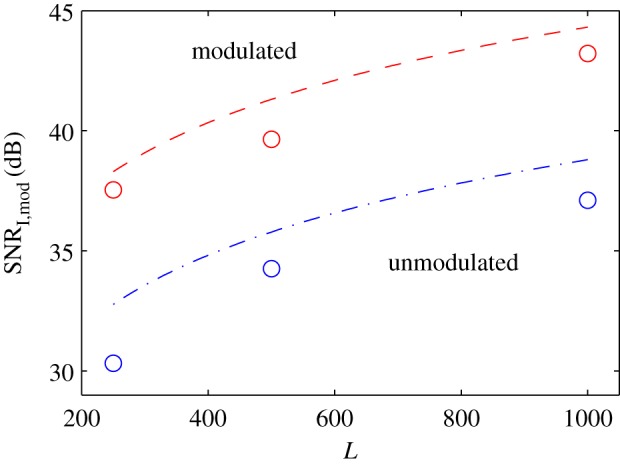
Predicted upper bound for the image SNR according to equations (3.8) and (3.9) which correspond to modulated and unmodulated sequences, respectively. The circle markers correspond to the experimental results–see experiment description for details. (Online version in colour.)

The SNR of the image which corresponds to the experiment of the wire underneath the probe was calculated in a region of the image where no reflections are expected, as in the previous section. The resulting SNR for *L*∈{250, 500, 10^3^} is shown in figure 14 using circle markers. Blue markers are used for the cases where the sequences are unmodulated, i.e. the received sequence are cross-correlated with sequences whose burst is an up-sampled delta function, and red marker are used when the sequences are modulated, i.e. the received sequences are cross-correlated with the actual transmitted ones. Experimental results correspond well with the predicted values using equations (3.8) and (3.9). In this particular example, matched filtering (modulation) produces an extra 6-dB SNR increase. The SNR input, SNR_in_, was calculated by averaging 10^3^ signals corresponding to an element right above the wire, finding the ratio between the maximum value of the echo and the variance where just noise exists and then dividing the result by 10^3^; this yielded SNR_ in_ ≈ 3.5 dB.

Consider the set-up of figure 13, where sequences which have *L* = 10^3^ intervals are used resulting in an overall duration of 10 ms. The image SNR with respect to the strongest reflector is approximately 37 dB. If sequential excitation were to be used to obtain the same SNR, 9 averages per element would have been needed according to equation (3.4), which corresponds to a total of 144 transmissions. To obtain a penetration depth of 100 mm, a waiting time of approximately 133 μs is required between transmissions. This yields a total duration of 192 ms; one order of magnitude slower compared to the case where the sequences are employed.

## 6. Discussion

We have used simple, point-like reflectors in order to validate the theoretical predictions against simulations and experimental results. Experimental data were acquired using a novel fully binary array controller. Furthermore, it was demonstrated that the methodology holds in the presence of multiple reflectors. The examples presented show that the proposed methodology can be an order of magnitude faster than equivalent sequential excitation even with just a small number of elements (16) and relatively short sequences (*L* = 10^3^).

We have limited ourselves to simple set-ups in this paper due to the limited number of elements of our prototype. A fair comparison with state-of-the-art systems using more complex and realistic set-ups will require a fully binary system with hundreds of elements, but this will require further development of the probe, the electronics and the data processing system. Initially, a conventional probe, such as the one reported in [1] could be used with a scaled version of the proposed electronics and acquisition card. Ultimately, a higher degree of integration between the probe and the front-end electronics will be preferred as well as a dedicated platform for the digital acquisition cards directly connected to graphic processing units for on-the-fly processing. However, we argue that because of the linearity of the proposed methodology, the present results can be extrapolated to those more complex cases. Hereinafter, we discuss the potential of the proposed methodology and make a case for its future development.

Let us take the probe reported in [1] as an example. This probe consists of 1024 elements. Say, that each element is excited with three cycles and one guard interval that has a length equivalent to one cycle, all centred at 5 MHz. Also, say that the overall acquisition duration should correspond to 50 frames per second. This makes it possible to use sequences that have a length *L* = 25 000. According to equation (3.2), all these parameters would reduce the noise/interference of the sequences by 70 dB when the number of elements equals 1024. If after such a noise reduction the noise of the sequences still predominates over the sidelobes of the PSF, this will correspond to an image SNR of 70 dB. Moreover, a much lower excitation power needs to be used since the SNR at the input should be less than 5 dB. On the other hand, if the elements were to be excited one at a time without any type of coding, the frame rate would be at least 500 times slower; this is without resorting to time averages and conservatively assuming that more than 40 intervals per transmit interval are required. Using conventional orthogonal sequences, such as those reported in [47], will not significantly speed up the acquisition of the signals corresponding to all of the possible paths either, because the length of the sequences that can be excited are limited by the location of the closest reflector.

A central problem for ultrasonic phased arrays is that denser (two-dimensional matrix) arrays produce signals with lower SNR because of the element's smaller area. In those scenarios, the proposed set of sequences will perform optimally if SNR_ in_ < 10 dB (figure 5). Moreover, images with low signal-to-coherent-noise-ratio (less than 50 dB) can also be found in materials that exhibit significant grain noise [90–93] or strong speckle in tissue [81,94–97]. Given that this sort of spatial noise is uncorrelated with the sequences and the electronics noise, the proposed sequences will also perform optimally in those cases, and a system that has more transmit–receive elements may be preferred due to the extra spatial averaging power.

Another key drawback of large arrays is the high data throughput required when using conventional acquisition systems. For example, sampling *M* = 10^3^ elements at 70 MHz using 8-bit ADCs will require a data throughput of roughly 560 Gbit s^−1^, which is difficult to achieve with current technology. However, if binary quantization [78] is used, this rate can be reduced to 70 Gbit s^−1^, which is already reported elsewhere. For example, a system that comprises 128 channels, is sampled at 70 MHz, and uses 12-bit ADCs was reported in [22]; this will produce a data throughput of 108 Gbit s^−1^. A further advantage is that the proposed sequences can be transmitted continuously [65] and hence a pipeline-like architecture can be used to reduce post-processing latency.

## 7. Conclusion

This paper introduces a set of sequences for radar, sonar or general ultrasound applications that enables an array to achieve full matrix capture with only one firing of the elements. The sequences have receive intervals so that the overall length of the sequences, and therefore the SNR increase, is not limited by the location of the closest reflectors and data from every transmit–receive pair in the array can be acquired simultaneously. This solves a long-standing problem in coded excitation and instrumentation for large arrays. The proposed set of sequences is shown to be at least one order of magnitude faster than the sequential excitation of individual elements when the received signals have low SNR (typically below 10 dB).

Moreover, when binary quantization is used together with the proposed sequences in low-SNR (less than 5 dB) scenarios, the data throughput is reduced by roughly one order of magnitude without affecting the performance or linearity of the system. An acquisition architecture is proposed whereby each array element is fully controlled by one digital line. This potentially allows more transmit-receive pairs to be acquired at higher frame rates, which translates into higher resolution and contrast. The prototype presented can be used with a maximum sampling frequency of 125 MHz per channel and produces focused images, whose SNR exceeds 40 dB, at a repetition rate greater than 100 Hz. The image SNR increases with the total number of elements and, in principle, the total number of elements has no limit and no effect on the repetition rate. A 16-channel binary array controller was built to experimentally demonstrate this way of acquiring signals. While the presented approach is generally applicable to any application where waves are used to interrogate a medium (e.g. radar, sonar, seismology, medical or industrial ultrasound), it is particularly suitable in applications that employ large (greater than 10^3^ elements) arrays that have low sensitivity. It is a suitable way to enable the design of scalable architectures for the next generation of arrays that have thousands of channels.

## Data accessibility

Readers who are interested in accessing data associated with this paper are referred to http://https://figshare.com/s/3e2cca2a9be478301563, DOI:10.6084/m9.figshare.7326707.

## Appendix A. Derivation of the sequence signal-to-noise ratio when using modulation and uneven paths

To obtain equation (3.8), let *w*_*ij*_ be the coefficients that correspond to the amplitude of the received echo from transmitter *i* and receiver *j*. Considering uneven paths, equation (3.1) can be rewritten for transmitter *i*′ and receiver *j*′ as

A 1 $${\text{SNR}}_{h}^{\prime }\approx \frac{L\left(1-p_{1}\right)w_{i^{\prime }j^{\prime }}^{2}}{\sum_{i=1}^{M}w_{ij}^{2}+(1/p_{1}{\text{SNR}}_{\mathrm{in}})},$$

where SNR_in_ is the input SNR with respect to the strongest echo. Combining all of the transmitters for receiver *j*′,

A 2 $${\text{SNR}}_{h}^{\prime \prime }\approx \frac{L\left(1-p_{1}\right){\left(\sum_{i=1}^{M}w_{ij^{\prime }}\right)}^{2}}{M\sum_{i=1}^{M}w_{ij}^{2}+(M/p_{1}{\text{SNR}}_{\mathrm{in}})}.$$

The right-hand term of the denominator, which simulates the receiver noise, is not affected in this process.

To include the effect of the modulation, assume that the sequences are up-sampled to match the burst length, that the modulated burst has variance *σ*^2^_*b*_ and mean zero, and that the received sequences are correlated with the up-sampled but unmodulated sequences. This process can be understood as a shifted combination of the transmitted sequence weighted by the burst samples. Since only the peak of the burst is of interest in the numerator, the weighting has no effect on it. Only the transmitted sequences are modulated, which has no effect on the right-hand term of the denominator either. Hence, the result of the modulation on transmission is simply the variance *σ*^2^_*b*_ multiplying the left-hand term of the denominator. Finally, by combining all of the receiver elements *j*, equation (3.8) is obtained.
